# A Global View of Transcriptome Dynamics during *Sporisorium scitamineum* Challenge in Sugarcane by RNA-seq

**DOI:** 10.1371/journal.pone.0106476

**Published:** 2014-08-29

**Authors:** Youxiong Que, Yachun Su, Jinlong Guo, Qibin Wu, Liping Xu

**Affiliations:** Key Laboratory of Sugarcane Biology and Genetic Breeding, Ministry of Agriculture, Fujian Agriculture and Forestry University, Fuzhou, Fujian, China; Institute of Crop Sciences, China

## Abstract

Sugarcane smut caused by *Sporisorium scitamineum* is a critical fungal disease in the sugarcane industry. However, molecular mechanistic studies of pathological response of sugarcane to *S. scitamineum* are scarce and preliminary. Here, transcriptome analysis of sugarcane disease induced by *S. scitamineum* at 24, 48 and 120 h was conducted, using an *S. scitamineum*-resistant and -susceptible genotype (Yacheng05-179 and “ROC”22). The reliability of Illumina data was confirmed by real-time quantitative PCR. In total, transcriptome sequencing of eight samples revealed gene annotations of 65,852 unigenes. Correlation analysis of differentially expressed genes indicated that after *S. scitamineum* infection, most differentially expressed genes and related metabolic pathways in both sugarcane genotypes were common, covering most biological activities. However, expression of resistance-associated genes in Yacheng05-179 (24–48 h) occurred earlier than those in “ROC”22 (48–120 h), and more transcript expressions were observed in the former, suggesting resistance specificity and early timing of these genes in non-affinity sugarcane and *S. scitamineum* interactions. Obtained unigenes were related to cellular components, molecular functions and biological processes. From these data, functional annotations associated with resistance were obtained, including signal transduction mechanisms, energy production and conversion, inorganic ion transport and metabolism, and defense mechanisms. Pathway enrichment analysis revealed that differentially expressed genes are involved in plant hormone signal transduction, flavonoid biosynthesis, plant-pathogen interaction, cell wall fortification pathway and other resistance-associated metabolic pathways. Disease inoculation experiments and the validation of *in* *vitro* antibacterial activity of the chitinase gene *ScChi* show that this sugarcane chitinase gene identified through RNA-Seq analysis is relevant to plant-pathogen interactions. In conclusion, expression data here represent the most comprehensive dataset available for sugarcane smut induced by *S. scitamineum* and will serve as a resource for finally unraveling the molecular mechanisms of sugarcane responses to *S. scitamineum*.

## Introduction

Sugarcane (*Saccharum officinarum*) is an important sugar crop, and disease within this commodity affects cane yield and sugar content. Sugarcane smut, or sugarcane whip smut, is an airborne fungal disease first discovered in South Africa’s Natal in 1877 [1]. The disease commonly manifests after infection with *Sporisorium scitamineum*, presenting as a black growth from the tip (“smut whip”) of the diseased sugarcane stalk. Infected sugarcanes sprout early, and tiller more than normal with slender stems and leaves. Also, smut whips grow on tillers, reducing sugarcane yield and sugar quality. Currently, sugarcane smut has emerged as a globally important disease, and prevalence is increasing annually. When infection is severe, it can cause a 20–50% loss in sugarcane production [2], [3]. Thus, replacing susceptible with resistant cultivars is a cost-effective measure for controlling sugarcane smut [4].

Currently, studies of the molecular mechanisms of resistance to sugarcane smut are still few and only preliminary. Raboin’s group used amplified fragment length polymorphism (AFLP) markers to analyze genetic maps of hybrids of resistant cultivar R570 and susceptible cultivar MQ76/53 [5]. Xu and colleagues developed random amplified polymorphic DNA (RAPD) markers for genes associated with resistance to sugarcane smut [6]. Thokoane and Rutherford applied cDNA-AFLP to investigate differentially expressed genes in sugarcane exposed to *S. scitamineum.* Sequence homology analysis revealed that with *S. scitamineum* stress, resistant cultivars differentially expressed putative serine/threonine protein kinase, chitin receptor kinase and long terminal repeat retrotransposon (LTR). In addition, 7 days after *S. scitamineum* infection, expression of phenylpropanoid, flavonoid genes and chitinase protein family members were induced [7]. Heinze and colleagues analyzed different gene sequences expressed in sugarcane after *S. scitamineum* infection, reporting that these genes involved transcription factors and signal receptors associated with disease resistance and proteases associated with the phenylpropane-flavonoid metabolic pathway [8]. Borrás-Hidalgo’s laboratory used a cDNA-AFLP technique for screening and obtained 62 genes that were differentially expressed after sugarcane infection with *S. scitamineum*. Among these, expression of 10 genes was down-regulated and 52 was up-regulated and of these, 19 were associated with defense and signal transduction. For example, sugarcane genes encoding nucleotide binding site-leucine-rich repeats, a nucleotide-binding site and a leucine-rich region (NBS-LRR), protein kinases and proteins associated with auxin and ethylene signaling pathways were found to be important to sugarcane smut resistance stability [9]. Que et al. applied both cDNA-AFLP and silver staining methods, and obtained 136 transcript-derived fragments (TDFs) differentially expressed in *S. officinarum* in response to *S. scitamineum* infection. Of these 40 TDFs (including 34 newly induced TDFs and 6 significantly up-regulated TDFs) were sequenced and data were confirmed using reverse transcription PCR (RT-PCR) [10].

Wu and colleagues applied a Solexa high-throughput sequencing technique to analyze differential gene expression after *S. scitamineum* infection, and obtained 2,015 differentially expressed sequence tags. Among these, 1,125 up-regulated and 890 down-regulated ESTs were identified, including 3 up-regulated ESTs associated with the MAPK signaling cascade pathway [11]. Que et al. examined sugarcane smut-resistant cultivar NCo376 and susceptible cultivar F134 using differential display PCR (DDRT-PCR) and identified 7 differentially expressed genes after *S. scitamineum* inoculation, and RT-PCR was applied to measure gene expression patterns in roots, stems and leaves after *S. scitamineum*, salicylic acid (SA) or hydrogen peroxide stress [12]. Su and colleagues used infected sugarcane buds to clone pathogenicity-associated β-1,3-glucanase genes *ScGluA* and *ScGluD* [13] with real-time quantitative PCR (RT-qPCR) examined gene expression, reporting that TDFs of target genes *ScGluA* and *ScGluD* were up-regulated under the stress of *S. scitamineum*. Moreover, compared to the susceptible cultivar, gene up-regulation in the resistant cultivar were faster, longer-lasting, and occurred in response to SA, methyl jasmonate (MeJA) or abscisic acid (ABA) induction, as well as NaCl or CdCl_2_ stress. Different expression patterns of *ScGluA* and *ScGluD* genes under biotic and abiotic stresses were also documented. Que and co-workers applied two dimensional electrophoresis (2-DE) to measure protein expression of sugarcane after *S. scitamineum* inoculation [14]. Using matrix-assisted laser desorption/ionization time of flight mass spectrometry (MALDI-TOF-TOF/MS), 23 differentially expressed proteins were identified and bioinformatic analysis revealed that 20 of these proteins were associated with photosynthesis, signal transduction or disease resistance and 3 proteins had an uncertain function. It can be deduced that, after *S. scitamineum* infection, various disease-resistant pathways are activated in sugarcane, and studies suggest that sugarcane and *S. scitamineum* interactions involve complex biological processes. Further in-depth research is needed to study the mechanism behind these observations.

RNA-Seq is an emerging transcriptomic technology utilizing high-throughput sequencing to analyze tissue or cell cDNA libraries obtained via reverse transcription of total RNA. After counting read numbers, RNA expression alterations were calculated to identify new TDFs. Until now, many transcriptomic studies have been conducted on stressed plants and many pathogen stress-response genes have been identified from *Arabidopsis thaliana*, *Oryza sativa, Zea mays,* and *Triticum aestivum*, and pathogen resistance mechanisms have been explored. Wu and colleagues [15] used Solexa sequencing to analyze mixed *Vitis vinifera* leaf samples collected 4–8 days after *Plasmopara viticola* inoculation, and obtained 15, 249 differentially expressed candidate genes. Ward and co-workers [16] applied RNA-Seq to obtain transcriptome expression profiles of red raspberry cultivars resistant and susceptible to *Phytophthora rubi*. Data indicated that expression of genes associated with lignin synthesis and the citric acid cycle, as well as genes encoding pathogenesis-related proteins and WRKY family transcription factors were all increased. Strau and colleagues [17] applied RNA-Seq to isolate one *Xanthomonas vesicatoria*-resistant gene-Bs4C-from *Capsicum annuum* which can regulate AvrBs4, a transcription activator-like effector of *Xanthomonas.* Li’s laboratory [18] used Solexa sequencing to analyze a transcriptome from an early interaction between *O. sativa* and *Magnaporthe grisea*, to provide a basis for investigating genes encoding *M. grisea* effector proteins and their functions. Thus, high-throughput techniques to examine the response of sugarcane inoculated with *S. scitamineum* at the transcriptome level may reveal metabolic pathways and molecular regulation networks involved, as well as to define the characteristics of transcriptional regulation and identify key genes involved in sugarcane smut resistance.

In the present study, a *S. scitamineum*-resistant sugarcane genotype (Yacheng05-179) and a susceptible genotype (“ROC”22) were analyzed 24, 48 and 120 h after *S. scitamineum* inoculation, and Illumina RNA-Seq sequencing, bioinformatics and RT-qPCR, transcriptome expression was performed to identify differentially expressed genes and offered detail of how sugarcane responds to *S. scitamineum* stress.

## Materials and Methods

### Ethics Statement

We confirm that no specific permits were required for the described locations/activities. We also confirm that the field studies did not involve endangered or protected species.

### Plant Materials and Pathogen Inoculation

The source of *S. scitamineum* inoculum was collected from the most popular cultivar “ROC”22 in the Key Laboratory of Sugarcane Biology and Genetic Breeding, Ministry of Agriculture (Fuzhou, China), and stored at 4°C. Two cultivars of sugarcane, *S. scitamineum-*resistant Yacheng05-179 and -susceptible “ROC”22, were also maintained in our laboratory. Robust stems were collected from both genotypes after soaking in water for 24 h. Stems were placed in a light incubator (12-h light-dark cycle, 32°C) for germination. When buds grew to 2 cm, 5×10^6^/mL *S. scitamineum* spore suspension (containing 0.01% volume ratio of Tween-20) was used to inoculate the sugarcane buds via puncture. Control buds received water inoculations. Next, sugarcane stems were cultured at 28°C and (12-h light-dark cycle) [19]. At 24, 48 and 120 h after inoculation, five single buds were randomly selected from each group, and immediately fixed with liquid nitrogen before being stored at −80°C. Each experiment was repeated three times.

### Total RNA Extraction, Construction of cDNA Library and Illumina Sequencing

The above five buds from Yacheng05-179, 24 h after water inoculation (T1) and 24, 48 and 120 h after *S. scitamineum* inoculation (T2–T4), and “ROC”22, 24 h after water inoculation (T5) and 24, 48 and 120 h after *S. scitamineum* inoculation (T6–T8), were collected for total RNA extraction using Trizol reagent (Invitrogen, Shanghai, China), respectively. At least 20 µL extracted total RNA was then sent to Beijing Biomarker Technologies Inc. for cDNA library construction and Illumina sequencing (HiSeqTM 2000, Illumina Inc., San Diego, CA, USA).

### Basic Data Processing and Analysis

Raw reads (double-ended sequences) obtained from sequencing were evaluated and a unigene library for sugarcane was obtained. Based on this library, gene structure annotation, expression analysis and function annotations were performed. The subroutine Getorf in the EMBOSS software package (http://emboss.sourceforge.net/apps/cvs/emboss/apps/getorf.html/) was used to predict open reading frames (ORFs). Comparing T2 *vs.* T1, T3 *vs.* T1, T4 *vs.* T1, T6 *vs.* T5, T7 *vs.* T5 and T8 *vs.* T5, unigene expression in both cultivars 24, 48 and 120 h after *S. scitamineum* inoculation were conducted. IDEG6 software (http://telethon.bio.unipd.it/bioinfo/IDEG6/) was used for a generalized Chi-square test, and obtained P values were corrected for multiple hypotheses testing using a false discovery rate (FDR). After correction, unigenes with false discovery rate (FDR) no greater than 0.01 and reads per kb per million reads (RPKM) between samples of no less than 2 (fold-change (FD) ≥2) were considered to be differentially expressed genes.

For gene function annotation, obtained unigene sequences were annotated by searching in various protein databases, including the National Center for Biotechnology Information (NCBI) non-redundant protein (Nr) database, the NCBI non-redundant nucleotide sequence (Nt) database, Swiss-Prot, TrEMBL, Cluster of Orthologous Groups (of proteins) (COG), Gene Ontology (GO) and the Kyoto Encyclopedia of Genes and Genomes (KEGG). Annotation information of homologous genes in these databases was used to represent annotations of obtained unigenes. In addition, information for differentially expressed genes was collected from unigene annotations, and these genes were subjected to GO and KEGG significant enrichment analyses to identify biological functions and metabolic pathways in which these genes participate.

### Customized Data Analysis

To identify dynamic changes in differentially expressed genes in sugarcane after *S. scitamineum* stress, expression of infected cultivars and controls at different time points (two groups), and between different time points of the same cultivar (multiple groups) were analyzed. Data for gene roles were then analyzed.

Two-group analysis. Specifically, two-group analysis was used to study differentially expressed genes of both cultivars at 24, 48 and 120 h after *S. scitamineum* inoculation and corresponding controls at 24 h. Then, up/down-regulated genes were counted. Differentially expressed genes were subjected to COG functional annotation, GO classification analysis, and KEGG enrichment analysis, to obtain information about gene function and relevant regulation networks at different time points.

Multi-group analysis. To analyze differential gene expression of one genotype at different time points (multi-group analysis), genes with sustained differential expression in both genotypes collected 24 h after water inoculation (control) and 24, 48 and 120 h after *S. scitamineum* inoculation were investigated to find gene intersections among the four time points. Moreover, differentially co-expressed genes in both cultivars at the same time point, and differentially co-expressed genes in both cultivars at different time points were also counted. Comparisons of T1, T2 *vs.* T1, T3 *vs.* T1 and T4 *vs.* T1, or T5, T6 *vs.* T5, T7 *vs.* T5 and T8 *vs.* T5, which underwent classification analysis in both cultivars and dynamic gene expression patterns, were obtained. For certain dynamic expression patterns, GO significance analysis and pathway enrichment analysis were performed.

### The Role of Chitinase Genes in Response to Pathogen Infection

Based on the RNA-Seq data, the unigenes encoding sugarcane chitinases were differently expressed in sugarcane after inoculation with *S. scitamineum*. The chitinase gene *ScChi* (unigene ID: gi36003099) was cloned and identified. Expression profiles of *ScChi* during Yacheng05-179-*S. scitamineum* interaction and “ROC”22-*S. scitamineum* interaction at 0 h, 24 h, 48 h and 120 h, as well as mock plants were investigated by RT-qPCR. The *ScChi* transcript was calculated by subtracting mock plant expression from inoculated sample at each corresponding time points.

For transient expression of *ScChi* in *Nicotiana benthamiana*, we constructed an overexpression vector pCAMBIA 1301-*ScChi* to analyze its defense response. The *Agrobacterium strain* EHA105 carrying the recombinant vector was grown overnight in LB liquid medium containing 35 µg/mL rifampicin and 50 µg/mL kanamycin at 28°C. Culture cells were collected and resuspended in MS liquid medium containing 200 µM acetosyringone at OD_600_ = 0.8. Then, cells were infiltrated into eight-leaf stage-old *N. benthamiana* leaves. For comparison, the *Agrobacterium strain* containing the pCAMBIA 1301 vector alone was also transiently expressed in *N. benthamiana* leaves. The materials were incubated at 28°C for 24 h (16 h light/8 h darkness). Then a dilution (OD_600_ = 0.5) of *Fusarium solani* var. *coeruleum* or *Botrytis cinerea* suspended in 10 mmol/L MgCl_2_ was infiltrated into the main vein of the infected leaves. Tested plants were cultured at 28°C (16 h light/8 h darkness) for 20 d and photographed.

To validate antifungal activity, *N. benthamiana* plants were infected with *Agrobacterium strain* EHA105 carrying pCAMBIA 1301-*ScChi* or pCAMBIA 1301 vector by the leaf disc method. The initial transgenic *N. benthamiana* lines (T_0_) was selected with 35 mg/mL hygromycin and were further identified by PCR and RT-PCR. The mycelium of the *Fusarium solani* var. *coeruleum* was inoculated in the middle of the petri plates containing potato dextrose agar (PDA). Four days after inoculation, filter papers ∼1 cm distance from hyphae were filled with chitinase from three different T_0_ generation of *ScChi* transgenic *N. benthamiana* plants, and controls were filled with chitinase from the T_0_ generation of pCAMBIA 1301 transgenic *N. benthamiana* or untransgenic *N. benthamiana* plants, or 0.1 mol/L sodium acetate buffer (pH 5.0). The antibacterial effect was observed after cultivation at 28°C for 2 d.

### RT-qPCR Validation

To validate the reliability of differentially expressed genes obtained from Illumina RNA-Seq sequencing, six co-expressed, up-regulated genes from both cultivars: sugar cane_unigene_BMK.40387 (metacaspase-1-like, Q1), sugar cane_unigene_BMK.49302 (ribonuclease 3-like, Q2), sugar cane_unigene_BMK.51436 (pathogenesis-related protein PR-10, Q3), sugar cane_unigene_BMK.57924 (sucrose transporter SUT1, Q4), sugar cane_unigene_BMK.63074 (vacuolar amino acid transporter 1-like, Q5) and sugar cane_unigene_BMK.63784 (heat shock protein-like, Q6) were subjected to RT-qPCR. First-strand cDNAs (10-fold dilution) of sugarcane buds collected from both cultivars 24 h after water inoculation (control) and 24, 48 and 120 h after *S. scitamineum* inoculation were used as templates, and specific primers were designed according to differential gene sequences [20] (Table S1 in File S1). Glyceraldehyde 3-phosphate dehydrogenase (*GAPDH*) [21] served as the internal reference gene. SYBR Green staining was applied for RT-qPCR using the ABI 7500 fast real-time PCR system (Applied Biosystems, Foster, CA, USA). The total reaction volume was 25 µL, including 12.5 µL FastStart Universal SYBR Green PCR Master (ROX Medical, Shanghai, China), 0.4 µmol/L primer and 2.0 µL template. Reaction conditions were: 50°C, 2 min; 95°C, 10 min; 95°C, 15 s, 60°C, 1 min, and 40 cycles and three replicates were performed for each. PCR using distilled water as the template was used as a blank control. A 2^−ΔΔCt^ algorithm was applied for quantitative gene expression analysis [22].

## Results

### RNA-Seq Results

Illumina RNA-Seq of eight samples yielded 36.68 Gb of data and 181,603,016 read pairs. Trinity software was used to assemble data for T1–T8. Data indicate that the assembled eight-sample unigene library included 148,605 unigenes. Among these, 46,525 exceeded 500 bp, accounting for 31.31% of all unigenes, and 20,798 exceeded 1.0 kb, accounting for 14% of all unigenes.

Merged data were assembled and clustered according to similarity to 15,394 sugarcane unigene sequences downloaded from the National Center for Biotechnology Information (NCBI) website to construct the merged unigene database (Merge_Unigene) and for subsequent analyses. As shown in Table 1, compared to unigenes obtained via simple assembling, Merge_Unigene had greater gene comparison efficiency. Merge_Unigene contains 99,824 unigenes. Among these, 47,345 exceeded 500 bp, accounting for 47.43% of all unigenes in Merge_Unigene, and 22,091 exceeded 1.0 kb, accounting for 22.13% of all unigenes.

**Table 1:** Assembly results of sugarcane transcriptome using trinity software.

Length range	Unigene	Merge_Unigene
200 bp–300 bp	28,473 (30.85%)	27,684 (27.73%)
300 bp–500 bp	24,852 (26.93%)	24,795 (24.84%)
500 bp–1,000 bp	18,488 (20.03%)	25,254 (25.30%)
1,000 bp–2,000 bp	12,792 (13.86%)	14,383 (14.41%)
2,000+bp	7,692 (8.33%)	7,708 (7.72%)
Total number	92,297	99,824
Total length	70,251,430 bp	77,293,229 bp
N50 length	1,300 bp	1,143 bp
Mean length	761.1453 bp	774.2950 bp

Notes: N50 length is an indicator of measuring assembly effect, which is calculated by the accumulated length of the assembled fragments from long to short. When the sum is greater than or equal to 50% of the total length, the final accumulated fragment length is the N50 value. Mean length = for the average assembly length.

The 99,824 sugarcane unigenes were annotated by searching various protein databases, including Nr, Nt, Swiss-Prot, TrEMBL, COG, GO and KEGG. In total, transcriptome sequencing of all the eight samples revealed gene annotations of 65,852 unigenes.

### Differential Gene Analysis of Both Genotypes at Different Time Points (Two-group Analysis)

#### Screening Two-group Genes after *S scitamineum* Inoculation

Two-group analysis yielded data about differentially expressed sugarcane genes at different time points after *S. scitamineum* inoculation (see Table 2). After *S. scitamineum* induction, up-regulated genes exceeded down-regulated genes. Also, as *S. scitamineum* inoculation was extended, differentially expressed genes in “ROC”22 gradually increased. After 120 h interaction, induced differentially expressed genes in “ROC”22 were 1.85 times greater than those of Yacheng05-179 (incompatible interaction). Also, differentially expressed genes in Yacheng05-179 48 h after *S. scitamineum* inoculation were greater than that at 24 and 120 h after inoculation, and exceeded the number of differentially expressed genes in “ROC”22 at the same time point (48 h). Data suggest that after *S. scitamineum* stress, differential gene expression was induced in the smut-resistant cultivar (24 and 48 h) earlier than that in the smut-susceptible cultivar (120 h).

**Table 2:** Statistics of differentially expressed genes.

Combination	DEG set name	Up regulated	Down regulated	All DEG
T2 *vs.* T1	DR24	323	185	508
T3 *vs.* T1	DR48	520	230	750
T4 *vs.* T1	DR120	1,270	535	1,805
T6 *vs.* T5	DY24	536	219	755
T7 *vs.* T5	DY48	832	265	1,097
T8 *vs.* T5	DY120	727	250	977

Notes: T2 *vs.* T1, T3 *vs.* T1 and T4 *vs.* T1 represent the combination of “ROC”22 under *S. scitamineum* stress for 24, 48, or 120 h and “ROC”22 under sterile water stress after 24 h, respectively. T6 *vs.* T5, T7 *vs.* T5 and T8 *vs.* T5 refer to the combination of Yacheng05-179 under *S. scitamineum* stress for 24, 48, or 120 h and Yacheng05-179 under sterile water stress after 24 h, respectively. Unigenes with false discovery rate (FDR) no greater than 0.01 and reads per kb per million reads (RPKM) between samples of no less than 2 (fold-change (FD) ≥2) were considered to be differentially expressed genes (DEG).

#### Functional Annotation of Differentially Expressed Genes

COG functional annotation revealed that after *S. scitamineum* stress, in “ROC”22, 22 differentially expressed general function genes were predicted at 24 h, 47 at 48 h, and 174 at 120 h. Functional annotation information associated with smut resistance, such as signal transduction mechanisms (16 genes distributed at 24 h, 19 genes distributed at 48 h, and 88 genes distributed at 120 h), energy production and conversion (5, 11, and 28), inorganic ion transport and metabolism (2, 8, and 33), and defense mechanisms (0, 2, and 14) were observed. Table S2 in File S1 showed the GO classification of up/down regulated genes (p≤0.05) in “ROC”22 after *S. scitamineum* inoculation. Some differentially expressed genes appeared to be related to smut resistance, including metabolic process (299, 460, and 1176), response to stimulus (236, 353, and 958), biological regulation (218, 297, and 838), immune system process (58, 80, and 213), and antioxidant activity (9, 15, and 21).

COG analysis of genes in Yacheng05-179 after *S. scitamineum* inoculation revealed that differentially expressed general function genes predicted at 24, 48 and 120 h were 61, 102 and 80, respectively. We also found some functional annotation information associated with smut resistance which involved in signal transduction mechanisms (21 genes distributed at 24 h, 40 genes distributed at 48 h, and 26 genes distributed at 120 h), energy production and conversion (17, 27, and 32), inorganic ion transport and metabolism (10, 24, and 19), and defense mechanisms (3, 7, and 3). The differentially expressed genes distributed to the metabolic process (505, 758, and 634), response to stimulus (400, 570, and 473), biological regulation (351, 511, and 424), immune system process (61, 102, and 72), and antioxidant activity (9, 18, and 19) were found according to GO analysis (Table S3 in File S1).

Data for functional annotation of differentially expressed genes obtained from two-group analysis reveal the overall transcriptome of sugarcane in response to *S. scitamineum* infection. TDF expression involves all aspects of biological activity. As the time of *S. scitamineum* inoculation extended, differentially expressed genes in “ROC”22 gradually increased. In addition, differentially expressed resistance-associated genes in Yacheng05-179 48 h after *S. scitamineum* inoculation were higher than those at 24 and 120 h after inoculation, and were also greater than differentially expressed genes with the corresponding COG function and GO classification in the susceptible cultivar “ROC”22 at 24 and 48 h. However, at 120 h, differentially expressed resistance-associated genes in the susceptible cultivar exceeded those in the resistant cultivar, suggesting that both cultivars have specific responses to *S. scitamineum*, and that the timing of induced gene expression in the smut-resistant cultivar was earlier.

### Differential Gene Analysis of a Sugarcane Genotype at Different Time Points (Multi-group Analysis)

Here, both cultivar controls and samples at 24, 48 and 120 h after *S. scitamineum* inoculation were subjected to multi-group differential analysis to identify differentially expressed genes in sugarcane associated with response to *S. scitamineum* infection.

#### Analysis of Differentially Co-expressed Genes at the Same Time Point in Both Genotypes

**Figure 1:**
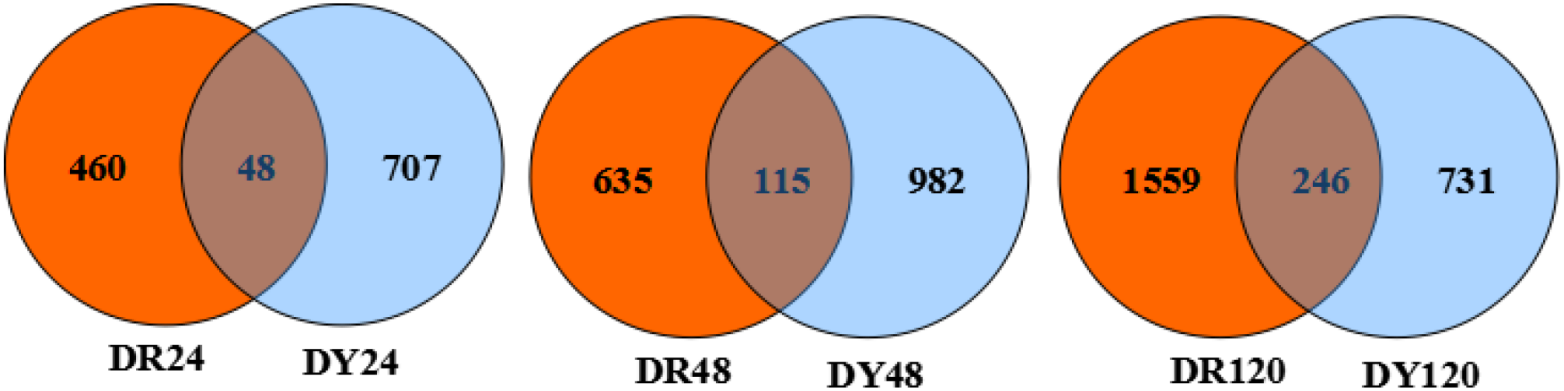
Venn diagram of differentially co-expressed genes in both sugarcane cultivars after *S. scitamineum* inoculation at the same time points. DR24, DR48 and DR120 denote differentially expressed gene sets obtained from “ROC”22 samples at 24, 48, and 120 h after *S. scitamineum* inoculation compared to controls 24 h after water inoculation, respectively; DY24, DY48 and DY120 denote differentially expressed gene sets obtained from Yacheng05-179 samples at 24, 48 and 120 h after *S. scitamineum* inoculation compared to control sample 24 h after water inoculation, respectively. All DEGs: differentially expressed genes.

Differentially expressed gene sets at the same time points of both cultivars (DR24–DY24, DR48–DY48 and DR120–DY120) were analyzed (see Fig. 1). At 24 h after *S. scitamineum* inoculation, 48 differentially co-expressed genes were identified in both cultivars (38 up-; 3 down-regulated). At 48 h, 115 differentially co-expressed genes were identified (103 up-; 5 down-regulated). Finally, at 120 h after inoculation, 246 differentially co-expressed genes were identified (218 up-; 18 down-regulated). Overall, after *S. scitamineum* infection of both cultivars, with increasing time, the number of induced differentially co-expressed genes increased.

#### Analysis of Sustained Differentially Expressed Genes in Both Genotypes Across Different Time Points

**Figure 2:**
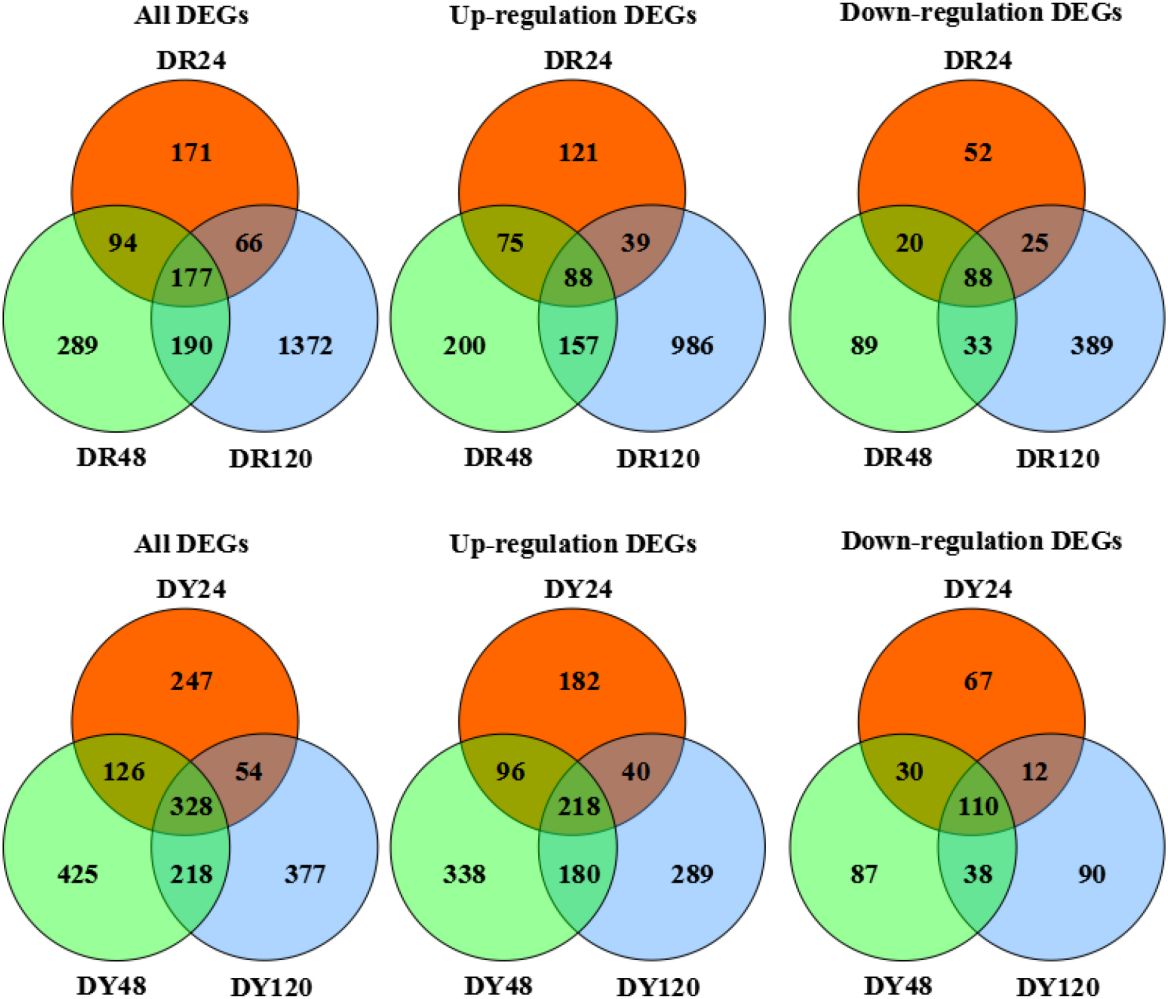
Venn diagram of differentially co-expressed genes in both sugarcane cultivar after *S. scitamineum* inoculation at different time points. DR24, DR48 and DR120 denote differentially expressed gene sets obtained from “ROC”22 samples at 24, 48 and 120 h after *S. scitamineum* inoculation compared to control sample at 24 h after water inoculation, respectively; DY24, DY48 and DY120 denote differentially expressed gene sets obtained from Yacheng05-179 samples at 24, 48 and 120 h after *S. scitamineum* inoculation compared to control sample at 24 h after water inoculation, respectively; All DEGs, all differentially expressed genes; Up-regulation DEGs, up-regulated genes; Down-regulation DEGs, down-regulated genes.

Differentially expressed gene sets at different time points of the same cultivar (DR24–DR48–DR120 h or DY24–DY48–DY120) were analyzed (see Fig. 2) and indicate that in “ROC”22 177 genes had sustained differential expression at 24, 48 and 120 h after inoculation (88 up-; 88 down-regulated). At 24 h 171 specific differentially expressed genes were identified (121 up-; 52 down-regulated). At 48 h, 289 were identified (200 up-; 89 down-regulated), and at 120 h, 1372 were identified (986 up-; 389 down-regulated). In Yacheng05-179, 328 genes had sustained differential expression at 24, 48 and 120 h after inoculation (218 up-; 110 down-regulated). At 24 h 247 specific differentially expressed genes were identified (182 up-; 67 down-regulated). At 48 h, 425 were identified (338 up-; 87 down-regulated) and at 120 h, 377 were identified (289 up-; 90 down-regulated). Thus from 24 to 120 h after *S. scitamineum* infection, continuously differentially expressed genes in Yacheng05-179 is greater than those in “ROC”22 (1.85 times more; 2.48 times more for up-regulated genes and 1.25 times more for down-regulated genes). Genes with sustained differential expression in both cultivars are depicted in Fig. 2.

#### Analysis of Genes with Sustained Differential Co-expression in Both Cultivars at Different Time Points

**Figure 3:**
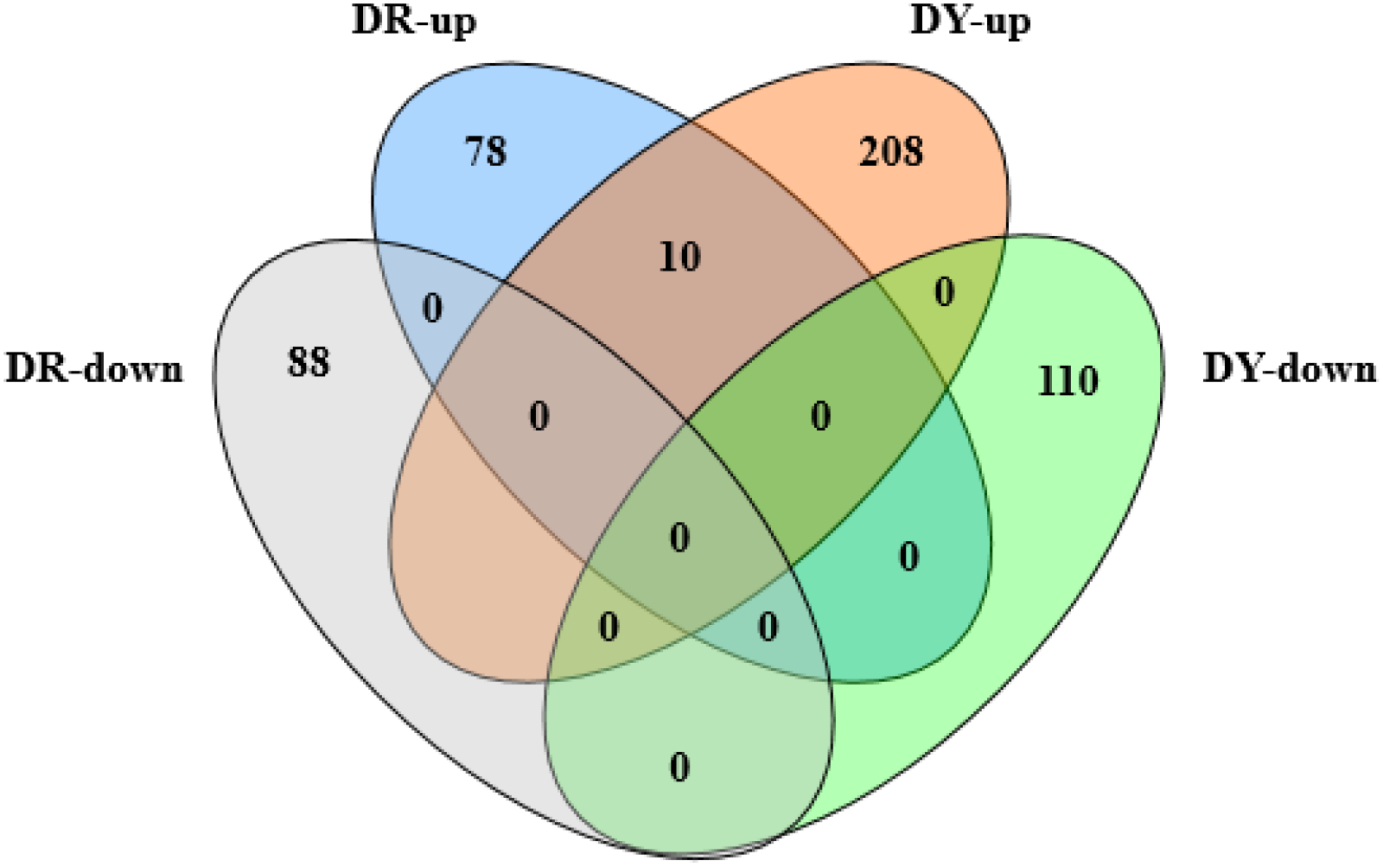
Venn diagram showing the number of genes with sustained differential co-expression between both sugarcane cultivars. DR-up and DR-down denote continuously up-regulated/down-regulated gene sets in “ROC”22 samples at 24, 48 and 120 h after *S. scitamineum* inoculation compared to control sample 24 h after water inoculation, respectively; DY-up and DY-down denote continuously up-regulated/down-regulated gene sets in Yacheng05-179 samples at 24, 48 and 120 h after *S. scitamineum* inoculation compared to control sample 24 h after water inoculation, respectively.

Differential genes with sustained expression in both cultivars at all time points were analyzed (see Fig. 3) and data suggest that the co-expressed, up-regulated genes are associated with plant resistance, and can be used as candidate resistance genes in future studies.

#### Analysis of Dynamic Gene Expression Pattern of Both Genotypes at Different Time Points

**Figure 4:**
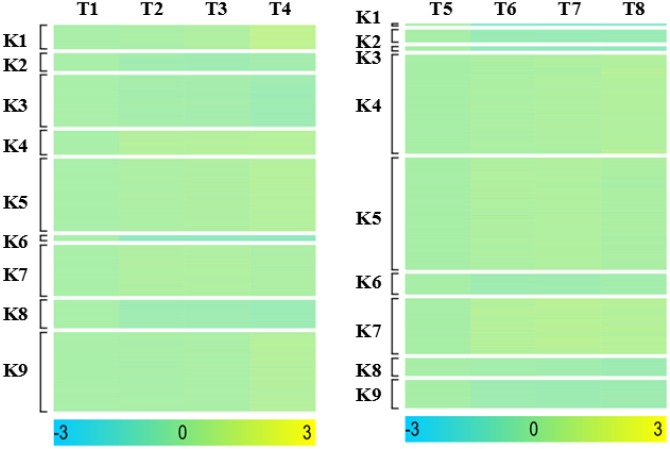
Cluster heatmap of expression patterns of differentially co-expressed genes in both sugarcane cultivars at different time points after *S. scitamineum* inoculation. T1, T2, T3 and T4 denote “ROC”22 at 24 h after water inoculation, and at 24, 48 and 120 h after *S. scitamineum* inoculation, respectively; T5, T6, T7 and T8 denote Yacheng05-179 at 24 h after water inoculation, and at 24, 48 and 120 h after *S. scitamineum* inoculation, respectively; k1∼k9 indicate nine distinct expression patterns of differentially co-expressed genes.

**Figure 5:**
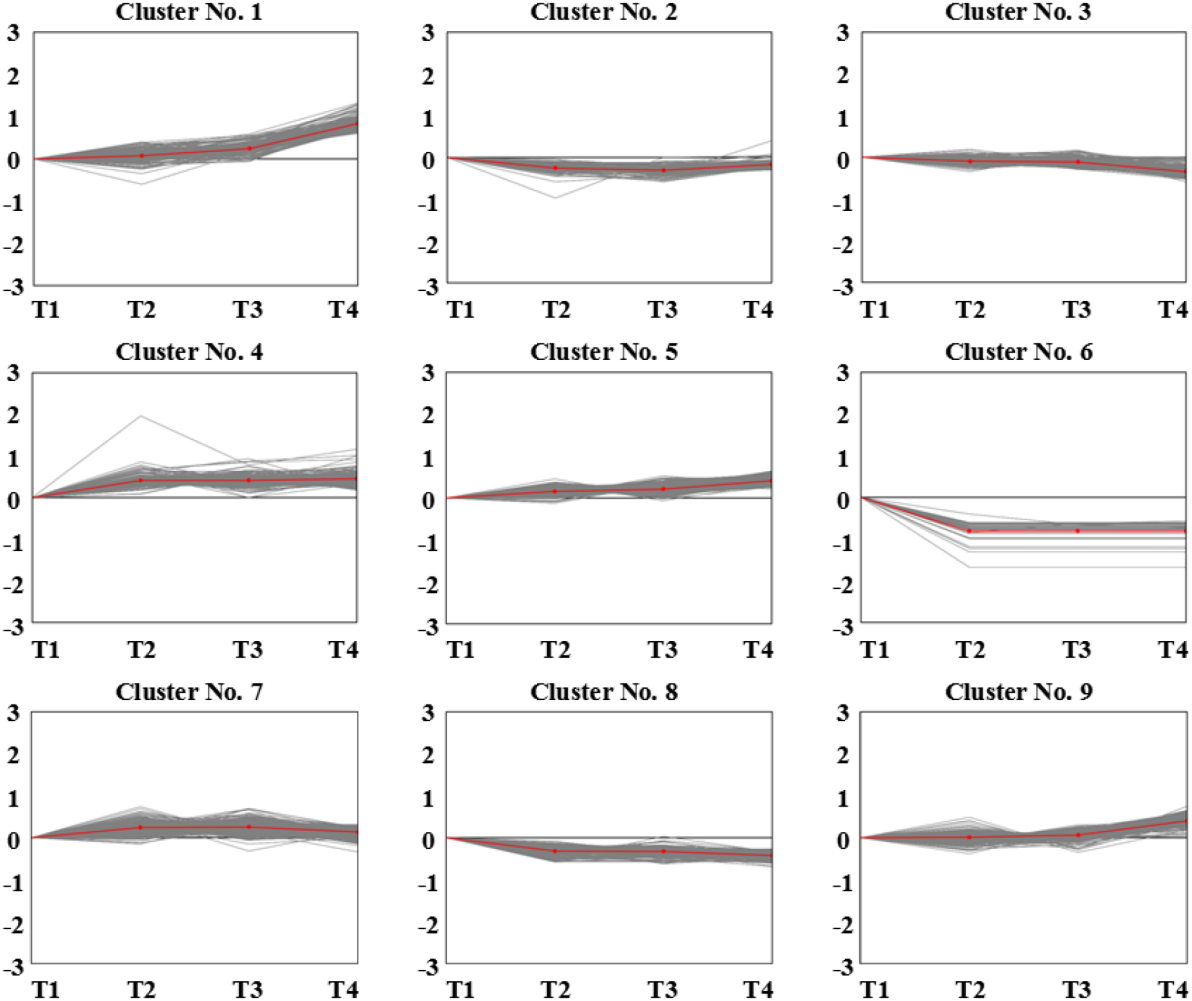
Dynamic expression models of differentially expressed genes in “ROC”22 after *S. scitamineum* inoculation (T1→T2→T3→T4). T1, T2, T3 and T4 denote “ROC”22 at 24 h after water inoculation, and at 24, 48 and 120 h after *S. scitamineum* inoculation, respectively.

**Figure 6:**
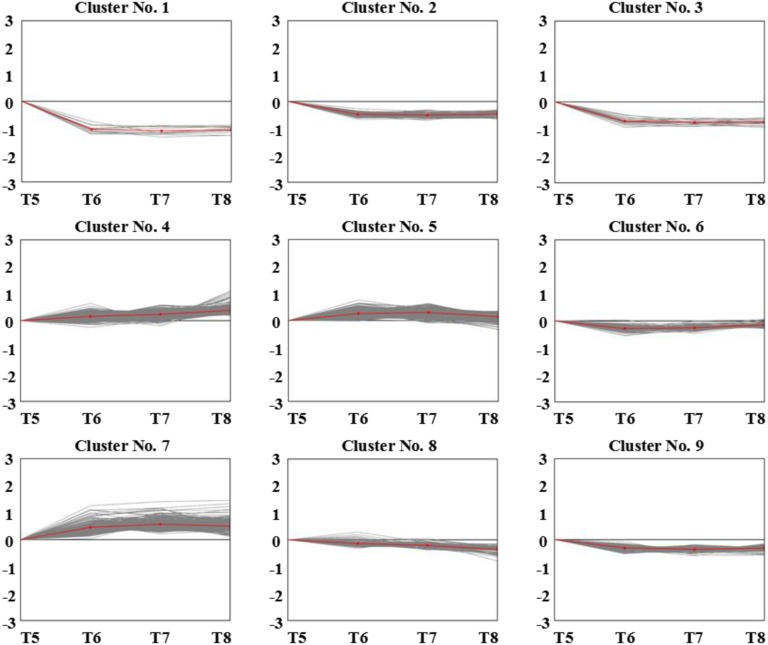
Dynamic expression models of differentially expressed genes in Yacheng05-179 after *S. scitamineum* inoculation (T5→T6→T7→T8). T5, T6, T7 and T8 denote Yacheng05-179 at 24 h after water inoculation, and at 24, 48 and 120 h after *S. scitamineum* inoculation, respectively.

Pathogen invasion into the host cell is a dynamic process and its growth and development in the host is a prerequisite for causing plant disease. During the invasion process, differentially expressed genes associated with disease resistance undergo changes in expression and genes with the same expression pattern for the same biological activity usually have similar functions. Here, we studied dynamic gene expression in both cultivars at different time points, and multi-group expression pattern clustering analysis was performed. After statistical analysis, cluster heatmaps of the dynamic expression patterns of differentially co-expressed genes in “ROC”22 or Yacheng05-179 at different time points (control→24 h→48 h→120 h, i.e. T1→T2→T3→T4 or T5→T6→T7→T8) (Fig. 4) and different dynamic expression models (Figs. 5 and 6) were plotted. As illustrated in Tables 3 or 4, in the four treated samples, 9 dynamic expression patterns of differentially expressed genes were identified. Combined with our knowledge about biological processes that *S. scitamineum* spores undergo after inoculation, we obtained three models of distinct and notable dynamic expression patterns of (0, 1, 2, 3), (0, −1, −2, −3) and (0, −1, −1, −1) in both genotypes.

**Table 3:** Dynamic expression patterns of differentially expressed genes in “ROC”22 after *S. scitamineum* inoculation.

Cluster No.	T1	T2	T3	T4	Gene Number
1	0	1	2	3	162
2	0	**−**2	**−**3	**−**1	118
3	0	**−**1	**−**2	**−**3	344
4	0	1	1	1	160
5	0	1	1	2	482
6	0	**−**1	**−**1	**−**1	39
7	0	2	2	1	339
8	0	**−**1	**−**1	**−**2	188
9	0	0	1	2	527

Notes: 0, 1, 2, 3, **−**1, **−**2 and **−**3 do not refer to the actual expression of the differentially expressed genes, but for the classification mark of gene dynamic changes. Gene numbers represent the actual number of dynamic expression patterns of differentially expressed genes. T1, “ROC”22 sample under sterile water stress after 24 h; T2–T4, “ROC”22 sample under *S. scitamineum* stress for 24, 48, and 120 h, respectively.

**Table 4:** Dynamic expression patterns of differentially expressed genes in Yacheng05-179 after *S. scitamineum* inoculation.

Cluster No.	T5	T6	T7	T8	Gene Number
1	0	**−**1	**−**3	**−**2	12
2	0	**−**1	**−**2	**−**1	64
3	0	**−**1	**−**1	**−**1	22
4	0	1	2	3	497
5	0	2	3	1	564
6	0	**−**3	**−**2	**−**1	104
7	0	1	3	2	280
8	0	**−**1	**−**2	**−**3	90
9	0	**−**1	**−**2	**−**1	142

Notes: 0, 1, 2, 3, −1, −2 and −3 do not refer to the actual expression of the differentially expressed genes, but for the classification mark of gene dynamic changes. Gene numbers represent the actual number of dynamic expression patterns of differentially expressed genes. T5, Yacheng05-179 sample under sterile water stress after 24 h; T6–T8, Yacheng05-179 samples under *S. scitamineum* stress for 24, 48, and 120 h, respectively.

In the pattern of (0, 1, 2, 3), Cluster No. 1 in “ROC”22 and Cluster No. 4 in Yacheng05-179, expression of differentially expressed genes continuously increased as inoculation time increased, and peaked at 120 h. The sustained accumulation of this gene type in response to *S. scitamineum* stress suggests important biological significance. Based on the different top 10 GO classifications, the number of differentially co-expressed genes in the resistant Yacheng05-179 cultivar was greater than that in “ROC”22 (Table 5). As for the pattern of (0, −1, −2, −3), Cluster No. 3 in “ROC”22 and Cluster No. 8 in Yacheng05-179, expression of differentially expressed genes continuously decreased as inoculation time increased, and were minimal at 120 h of plant-pathogen interaction. In the pattern of (0, −1, −1, −1), Cluster No. 6 in “ROC”22 and Cluster No. 3 in Yacheng05-179, differentially expressed genes were expressed at a low levels in controls, and maintained even lower expression at different time points after *S. scitamineum* stress, suggesting sustained down-regulation of these gene types after pathogen induction. Also, based on the different top 10 GO classifications in these two dynamic expression patterns, the number of differentially co-expressed genes in resistant cultivar were less than that in the susceptible one (Table 5). It is interesting that the genes of Cluster No. 3 in the susceptible cultivar “ROC”22 were continuously down-regulated after *S. scitamineum* inoculation, and these were involved in the DNA replication pathways (14), pyrimidine metabolism (8), purine metabolism (7), ribosome (6), nucleotide excision repair (6), mismatch repair (6), homologous recombination (6), ubiquitin mediated proteolysis (5), glutathione metabolism (3) and base excision repair (3).

**Table 5:** Analysis of GO classifications involving differentially co-expressed genes in three models of distinct and notable dynamic expression patterns in both sugarcane genotypes.

GO classifications	Pattern (0, 1, 2, 3)		Pattern (0, −1, −2, −3)		Pattern (0, −1, −1, −1)	
	“ROC”22	Yacheng05-179	“ROC”22	Yacheng05-179	“ROC”22	Yacheng05-179
cell	110	372	284	65	21	10
cell part	115	379	285	65	22	10
organelle	103	340	281	64	20	10
organelle part	0	0	0	34	0	0
membrane	54	203	0	42	17	0
cellular process	91	324	267	59	20	11
cellular componentorganization or biogenesis	0	0	180	0	0	0
metabolic process	96	0	241	62	0	10
developmental process	0	317	165	0	0	7
response to stimulus	87	0	179	31	20	10
immune system process	0	246	0	0	15	0
biological regulation	54	210	199	0	19	10
multicellular organismal process	0	0	0	0	0	7
catalytic activity	72	202	0	48	0	0
binding	77	274	247	49	17	10
localization	0	0	0	0	16	0

### Analysis of Metabolic Pathways

Analysis of metabolic pathways in which differentially expressed genes may be involved or may participate are shown in Tables 6 and 7. After *S. scitamineum* inoculation in both cultivars, differential gene expression involved in resistance-associated metabolic pathways were induced (Tables 6, 7 and 8). In addition, as infection time increased (24 to 120 h), differentially expressed genes involved in metabolic pathways gradually increased. At 24, 48 and 120 h after infection, differentially expressed genes involved in metabolic pathways in “ROC”22 were 112, 142 and 287, respectively. Differentially expressed genes involved in metabolic pathways in Yacheng05-179 were 138, 217 and 202, respectively. Overall, after *S. scitamineum* inoculation, during early and middle stages of infection (24 and 48 h) differentially expressed genes involved in resistance-associated metabolic pathways in Yacheng05-179 were greater than those of the susceptible cultivar. Resistance-associated metabolic pathways of significant enrichment were also greater than those in “ROC”22. Thus, further exploration of differentially expressed genes in metabolic pathways is needed to understand the mechanism underlying sugarcane smut resistance.

**Table 6:** Analysis of pathways involving differentially expressed genes after *S. scitamineum* inoculation in “ROC”22.

Pathway	ko_id	T2 *vs.* T1		T3 *vs.* T1		T4 *vs.* T1	
		Cluster_frequency	P-value	Cluster_frequency	P-value	Cluster_frequency	P-value
Plant-pathogeninteraction	ko04626	7 out of 112 6.25%	0.0494	6 out of 142 4.24%	0.1354	4 out of 287 1.39%	0.8759
Phenylalanine metabolism	ko00360	3 out of 112 2.68%	0.3411	2 out of 142 1.41%	0.6382	5 out of 287 1.74%	0.3392
Phenylpropanoidbiosynthesis	ko00940	3 out of 112 2.68%	0.4184	2 out of 142 1.41%	0.7069	6 out of 287 2.09%	0.2714
Plant hormone signaltransduction	ko04075	3 out of 112 2.68%	0.9346	6 out of 142 4.22%	0.5623	8 out of 287 2.79%	0.8507
Flavonoid biosynthesis	ko00941	2 out of 112 1.79%	0.2076	3 out of 142 2.11%	0.0573	3 out of 287 1.05%	0.2081
Terpenoid backbonebiosynthesis	ko00900	1 out of 112 0.89%	0.7516	3 out of 142 2.11%	0.1702	2 out of 287 0.70%	0.7300
Peroxisome	ko04146	1 out of 112 0.89%	0.9322	2 out of 142 1.41%	0.7644	1 out of 287 0.35%	0.9935
Ribosome	ko03010	10 out of 112 8.93%	0.3227	8 out of 142 5.63%	0.6471	10 out of 287 3.48%	0.9534

Notes: T2 *vs.* T1, T3 *vs.* T1 and T4 *vs.* T1 =  combination of “ROC”22 sample under *S. scitamineum* stress for 24, 48, or 120 h and Yacheng05-179 under sterile water stress after 24 h, respectively.

**Table 7:** Analysis of pathways involving differentially expressed genes after *S. scitamineum* inoculation in Yacheng05-179.

Pathway	ko_id	T6 *vs.* T5		T7 *vs.* T5		T8 *vs.* T5	
		Cluster_frequency	P-value	Cluster_frequency	P-value	Cluster_frequency	P-value
Plant-pathogen interaction	ko04626	3 out of 138 2.17%	0.8646	10 out of 217 4.61%	0.0437	3 out of 202 1.49%	0.8842
Phenylalanine metabolism	ko00360	12 out of 138 8.70%	-	7 out of 217 3.23%	0.0486	8 out of 202 3.96%	0.0119
Phenylpropanoid biosynthesis	ko00940	12 out of 138 8.70%	0.0001	7 out of 217 3.23%	0.0844	8 out of 202 3.96%	0.0241
Plant hormone signal transduction	ko04075	8 out of 138 5.80%	0.5909	15 out of 217 6.91%	0.0463	9 out of 202 4.46%	0.5035
Flavonoid biosynthesis	ko00941	2 out of 138 1.45%	0.3429	2 out of 217 0.92%	0.3999	2 out of 202 0.99%	0.3647
Terpenoid backbone biosynthesis	ko00900	2 out of 138 1.45%	0.5882	3 out of 217 1.38%	0.3814	3 out of 202 1.49%	0.3380
Peroxisome	ko04146	0 out of 138 -	-	3 out of 217 1.38%	0.8038	2 out of 202 0.99%	0.9100
Ribosome	ko03010	0 out of 138 -	-	0 out of 217 -	-	1 out of 202 0.50%	1.0000

Notes: T6 *vs.* T5, T7 *vs.* T5 and T8 *vs.* T5 refer to the combination of Yacheng05-179 under *S. scitamineum* stress for 24, 48, or 120 h and “ROC”22 under sterile water stress after 24 h, respectively.

**Table 8:** Expression of resistance-related genes in sugarcane after *S. scitamineum* infection.

pathway	Gene	No. change	T2 *vs.* T1	T3 *vs.* T1	T4 *vs.* T1	T6 *vs.* T5	T7 *vs.* T5	T8 *vs.* T5
			No. 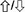	No. 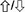	No. 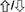	No. 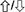	No. 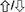	No. 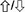
Plant hormone signal transduction	*PYR/PYL*	2	0/0	0/0	0/0	0/0	0/0	2/0
	*PP2C*	3	0/0	0/0	0/0	0/3	0/2	0/1
	*SnRK2*	3	0/0	1/0	1/0	0/0	0/1	0/0
	*JAZ*	7	3/0	3/0	1/0	2/0	5/0	3/0
	*MYC2*	1	0/0	0/0	0/0	1/0	1/0	0/0
Flavonoid biosynthesis	*PAL*	4	1/0	1/0	1/0	3/0	1/0	0/0
	*C4H*	4	1/0	1/0	1/0	1/0	2/0	2/0
	*4CL*	2	0/0	0/0	0/0	1/0	1/0	0/0
Plant-pathogen interaction	*Glucanase*	10	1/0	2/0	8/0	2/0	1/0	4/0
	*Chitinase*	26	0/3	4/5	15/3	11/0	7/0	3/0
	*Catalase*	1	0/0	1/0	1/0	0/0	1/0	1/0
Cell wall fortification pathways	*Syntaxin*	8	1/0	0/0	3/0	1/0	3/0	0/0
	*HRGP*	9	3/0	5/0	3/1	1/0	1/0	2/0
	*CER1*	1	0/0	0/0	1/0	0/0	0/0	0/0
Transcription factors	*MYB*	25	2/0	0/0	12/0	3/2	5/1	7/3
	*WRKY*	18	3/0	3/0	13/0	3/1	4/1	2/1
	*ERF*	18	6/0	2/1	2/1	8/1	8/0	9/0

Notes: T2 *vs.* T1, T3 *vs.* T1 and T4 *vs.* T1 refer to a combination of “ROC”22 under *S. scitamineum* stress for 24, 48, or 120 h and “ROC”22 under sterile water stress after 24 h, respectively. T6 *vs.* T5, T7 *vs.* T5 and T8 *vs.* T5 refer to the combination of Yacheng05-179 under *S. scitamineum* stress for 24 h, 48 h or 120 h and Yacheng05-179 under sterile water stress after 24 h, respectively.

### The Role of Chitinase Genes in Response to Pathogen Infection

**Figure 7:**
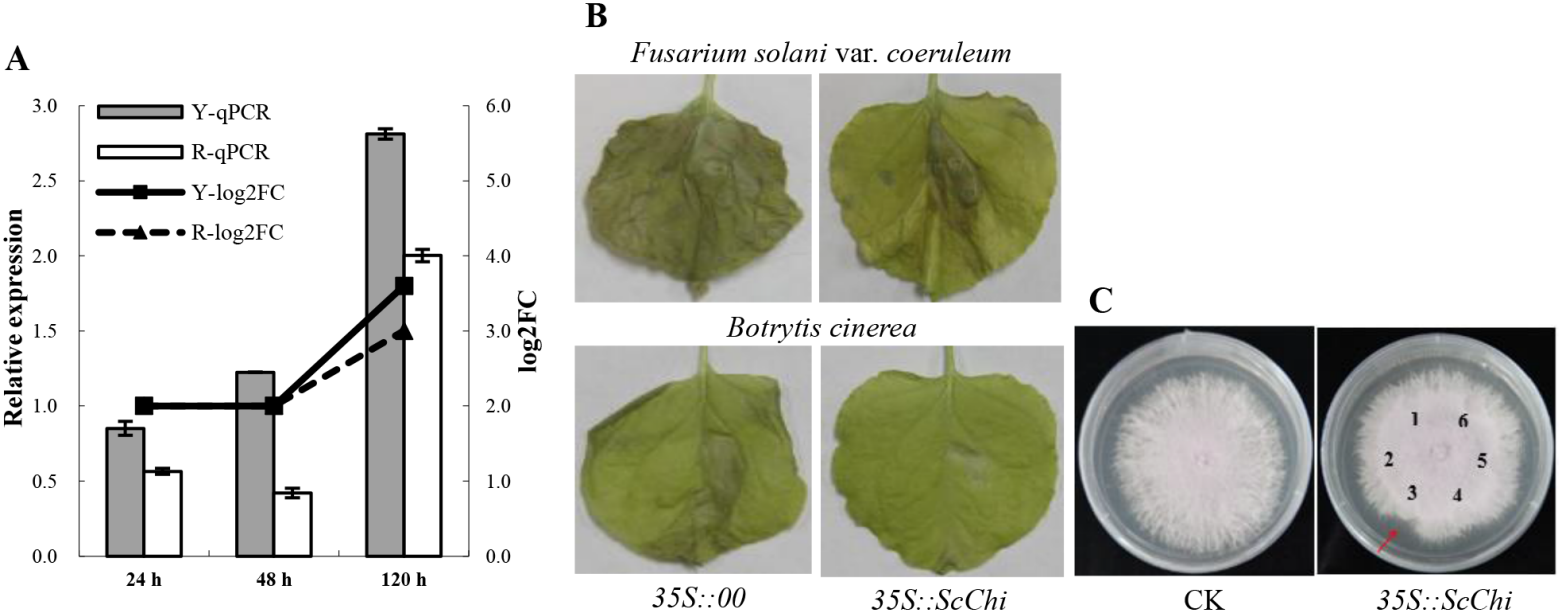
Analysis of the gene encoding sugarcane chitinase. (A) Transcript levels of *ScChi* during sugarcane-*Sporisorium scitamineum* interaction. The data of RT-qPCR was normalized to the *GAPDH* expression level. All data points (deduction its mock) are means ±SE (n = 3). Y, Yacheng05-179; R, “ROC”22. 24 h, 48 h and 120 h, sugarcane buds inoculation with *S. scitamineum* at 24 h, 48 h and 120 h, respectively; qPCR, detection results of real-time fluorescent quantitative PCR; log_2_FC, fold change of the differential expression of chitinase gene in the transcriptome. (B) The infection results of *Nicotiana benthamiana Fusarium solani* var. *coeruleum* and *Botrytis cinerea* by infiltrated with the *35S::ScChi*-containing *Agrobacterium* strain. The disease symptom was assessed 20 d after inoculation. (C) The antimicrobial action of chitinase (T_0_ generation of ScC*hi* transgenic *N. benthamiana*) on *Fusarium solani* var. *coeruleum.* CK, the control of normal culture on *Fusarium solani* var. *coeruleum*; *35S::ScChi*, the antimicrobial action of chitinase of T_0_ generation of *ScChi* transgenic *N. benthamiana*; 1∼3, chitinase from three different T_0_ generation of *ScChi* transgenic *N. benthamiana*, respectively; 4, chitinase from T_0_ generation of pCAMBIA 1301 transgenic *N. benthamiana*; 5, chitinase from untransgenic *N. benthamiana*; 6, 0.1 mol/L sodium acetate buffer (pH 5.0). Read arrow indicated the antibacterial effect.

Chitinases (EC 3.2.2.14), which can catalyze poly chitin are present in the cell walls of most fungi, and homologues in plant typical pathogenesis-related (PR) proteins. Our results indicated that 26 unigenes (gi36003099, Sugarcane_Unigene_BMK.51590, Sugarcane_Unigene_BMK.47839, Sugarcane_Unigene_BMK.34637, gi34957207, Sugarcane_Unigene_BMK.38981, gi35081719, gi35238203, Sugarcane_Unigene_BMK.38726, gi35992663, Sugarcane_Unigene_BMK.69934, Sugarcane_Unigene_BMK.49423, gi35980761, gi36002588, Sugarcane_Unigene_BMK.44826, Sugarcane_Unigene_BMK.56580, gi32815041, Sugarcane_Unigene_BMK.60969, Sugarcane_Unigene_BMK.40091, gi35045219, gi36066432, Sugarcane_Unigene_BMK.60821, gi36021860, Sugarcane_Unigene_BMK.48857, Sugarcane_Unigene_BMK.68059 and Sugarcane_Unigene_BMK.64954) encoding chitinases were differently expressed in sugarcane after inoculation with *S. scitamineum* (Table 8). The transcript of an acidic class III chitinase *ScChi* (gi36003099) was triggered during challenge with *S. scitamineum* in both resistant (Yacheng05-179) and susceptible (“ROC”22) cultivars, but gene expression was greater and maintained longer in the resistant cultivar (Fig. 7A). To determine whether *ScChi* (GenBank Accession No. KF664180) affects resistance to fungi, over-expressing pCAMBIA 1301-*ScChi* helped improve *N. benthamiana* in defending *Fusarium solani* var. *coeruleum* and *Botrytis cinerea* after inoculation for 20 d (Fig. 7B) which had significantly greater disease resistance than controls. Meanwhile, chitinase from plant 3 of the T_0_ generation of *ScChi* transgenic *N. benthamiana* could inhibit hyphal growth of *Fusarium solani* var. *coeruleum* (Fig. 7C).

### RT-qPCR Validation

**Figure 8:**
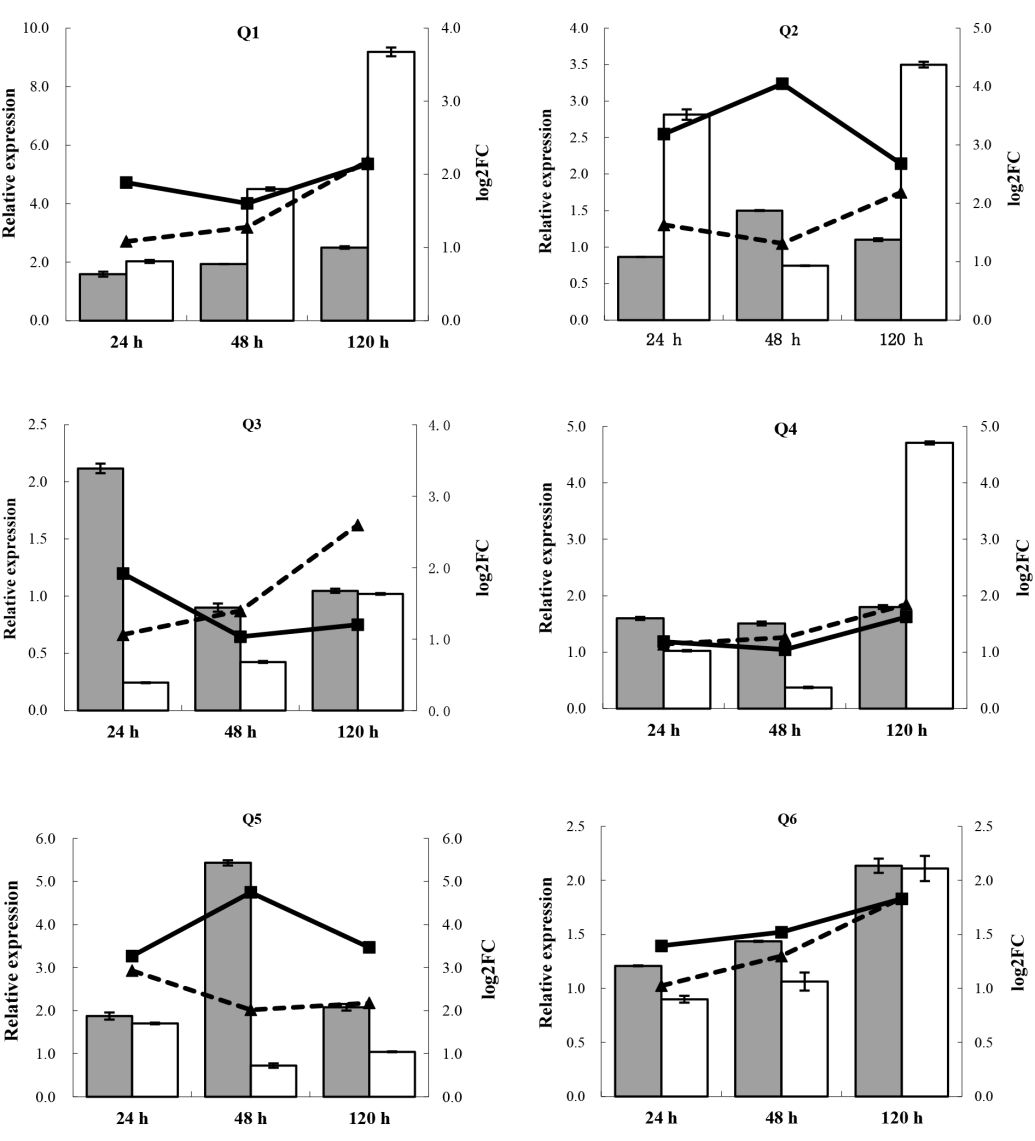
RT-qPCR validation of parts of differentially expressed genes identified by Illumina sequencing. Q1, metacaspase-1-like gene; Q2, ribonuclease 3-like gene; Q3, pathogenesis-related protein (PR-10) gene; Q4, sucrose transporter (SUT1) gene; Q5, vacuolar amino acid transporter 1-like gene; Q6, heat shock protein-like gene. Y, Yacheng05-179; R, “ROC”22; 24 h, 48 h and 120 h, sugarcane buds inoculation with *Sporisorium scitamineum* at 24 h, 48 h and 120 h, respectively; qPCR, detection results of real-time fluorescent quantitative PCR; log_2_FC, fold change of the differential expression genes in the transcriptome.

To validate sequencing reliability, 6 differentially co-expressed genes in both genotypes were subjected to RT-qPCR analysis (metacaspase-1-like gene (Q1), ribonuclease 3-like gene (Q2), pathogenesis-related protein (PR-10) gene (Q3), sucrose transporter (SUT1) gene (Q4), vacuolar amino acid transporter 1-like gene (Q5) and heat shock protein-like gene (Q6)). RT-qPCR results (Fig. 8) for these differentially expressed genes were similar to Illumina sequencing results, but bias in the degree of differential expression between the two data sets, likely because the sensitivity of Illumina sequencing is greater than that of RT-qPCR [23]. In general, RT-qPCR data depicted up/down regulation patterns of differential TDFs that were consistent with Illumina sequencing results, suggesting that Illumina data are relatively reliable.

## Discussion

### Application of Illumina RNA-Seq in Transcriptome Studies

With RNA-Seq technology, no prior assumption is needed to obtain transcriptional activity of tissues or cells under particular conditions (including coding and non-coding RNAs). Illumina technology can combine reads of different lengths, single and double-terminal sequencing, strand specificity, and obtain billions of reads. This method permits not only annotation of encoding simple sequence repeats (SSR) and single nucleotide polymorphisms (SNP), as well as identification of alternative splicing, but also allows discovery of new and rare TDFs, while identifying regulatory RNAs and determining the expression abundance of TDFs. Thus, accurate and complete gene function maps can be obtained. Transcriptome sequencing technology has widely used in many studies-such as bilberry fruit transcriptome library sequencing and investigating expression of genes associated with anthocyanin biosynthesis [24], transcriptome analysis of eucalyptus under scorch viral stress [23], and transcriptome analysis of interactions between tomato and powdery mildew [25].

Here, RNA-Seq of eight sugarcane samples yielded 36.68 GB data and 181,603,016 pairs of reads. By assembling data from each cultivar (Table 1), a Unigene library was constructed. Unigenes of sugarcane published online were merged with sequencing data to construct the Merge_Unigene library. In the Unigene library and the Merge_Unigene library, there are 148,605 and 99,824 unigenes, respectively but unigenes exceeding 500 bp and 1.0 kb in both libraries do not differ much. TDFs of rather low expression were also assembled relatively completely in Merge_Unigene. Thus, for subsequent analyses, the Merge_Unigene database was used. Searches indicate that gene numbers of *Z. mays*, *Sorghum bicolor*, and other species related to *S. officinarum* fall into the range of 30,000–40,000, a number similar to the number of relatively long unigenes (99,824) after merged assembling, suggesting a satisfactory outcome of merged assembling. Subsequent comparisons of unigenes with various databases showed that for most unigenes, especially those longer than 1.0 kb, annotation information on homologous sequences could be obtained. This also indicates the accuracy of the unigenes we obtained after assembling. The Merge_Unigene library constructed provided sufficient information for subsequent analysis and can be a reference for future sugarcane studies.

### Bioinformatic Annotation of Unigenes

Sugarcane is a highly heterozygous allopolyploid or highly heterozygous aneuploid crop and sugarcane hybrids have more than 120 chromosomes (genome sizes = 10 GB [26]). Whole genome sequencing has not been completed but the number of unigenes published by NCBI is 15,394. Here, we observed that the Merge_Unigene database had better comparison efficiency than the Unigene library; however, the comparison efficiency between the Merge_Unigene and *S. bicolor* unigene database was not high. Although *S. bicolor* and *S. officinarum* are related species, their gene sequences differ. Merging the *S. officinarum* unigene library with that of *S. bicolor* will introduce many unigenes that are useless to *S. officinarum*, and may likely affect subsequent analyses. Hence, the obtained *S. officinarum* library was not merged with the *S. bicolor* unigene library.

Bioinformatic annotations of the unigenes obtained 65,852 unigene annotations involved in cell parts, molecular functions and biological processes, among other functions. From this analysis, functional annotations associated with resistance were obtained (signal transduction mechanisms, energy production and conversion, inorganic ion transport and metabolism and defense mechanisms). Pathway enrichment analysis revealed that differentially expressed genes are involved in plant-pathogen interaction (ko04626), plant hormone signal transduction (ko04075), phenylalanine metabolism (ko00360), peroxisome (ko04146), flavonoid biosynthesis (ko00941), phenylpropanoid biosynthesis (ko00940), ribosome (ko03010) and other resistance-associated metabolic pathways. As illustrated in Table 2, after *S. scitamineum* induction, up-regulated genes in both cultivars exceeded down-regulated genes. In addition, as the *S. scitamineum* infection time was prolonged, differentially expressed genes gradually increased. Transcriptome sequencing analysis showed that most genes and pathways induced in both cultivars were similar. *S. scitamineum* activated multiple smut-resistance pathways, and differentially expressed genes were involved in defense response, signal transduction and other processes. The response to *S. scitamineum* involved almost all aspects of biological activities, suggesting the pathogen response is regulated by multi-gene networks, a finding consistent with previous data which suggest that after pathogens infect plants, many metabolic pathways are affected, and gene expression in the transcription network is disturbed [27]–[29].

What should also be stressed here is that, when comparing the samples at 48 h and 120 h post *S. scitamineum* inoculation with the samples at 24 h post water inoculation, they do have some differentially expressed genes which are involved in the senescing process. In the present study, as for this issue, firstly, only unigenes with no less than 2 (fold-change (FD) ≥2) were considered to be differentially expressed genes, which should largely decrease or even avoid the number of genes related to senescing process. Secondly, before we decide to further investigate the function of a certain differentially expressed gene, we need to confirm again that the differential expression is due to the challenged by *S. scitamineum*, but not only because of the senescing process, such as by RT-qPCR or Northern blot.

In the present studies, 33,972 unigenes (34.03%) were not annotated, likely due to incomplete whole genome sequencing of sugarcane, suggesting that a whole-genome database with functional annotations has not been established. Also, the number of sugarcane ESTs with known functions is limited and some short sequences obtained affected data comparisons. These unannotated unigenes may be new TDFs, and future experiments are needed to confirm this.

### Analysis of Resistance-Associated Metabolic Pathways and Genes

Mechanisms underlying plant resistance to unique pathogens are diverse involving many molecular processes that could be slow or weak in susceptible plants. Such molecular changes affect later alterations in plant appearance, physiology and biochemistry. Chief differences between resistant and susceptible plants can be found in the timing of host recognition of pathogen invasion, and the defense reaction speed and effectiveness. In susceptible plants, slow response and weaker defense signals allow pathogens to travel throughout and damage the plant [25]. How sugarcane and *S. scitamineum* interact at a molecular level is complex, so we investigated how two sugarcane cultivars confer resistance or susceptibility to *S. scitamineum* inoculation and whether this response is based on differential expression of genes involved with induction of resistance-associated metabolic pathways.

#### Plant Hormone Signal Transduction Pathways

Plant hormones are produced in response to environmental factors that regulate physiological reactions at low concentrations and include auxin, gibberellin acid (GA), cytokinin (CK), ABA, ethyne (ETH), SA, jasmonic acid (JA), brassinosteroid (BR) and polyamines. Plant hormones independently or collaboratively regulate plant growth, development and differentiation through cell division and elongation, differentiation of tissues and organs, sleep, seed germination, flowering and fruiting, aging and *in* *vitro* culture. Some plant hormones, such as SA, JA and ET which defend against pathogens have been well studied [30]. SA, JA and ET have been reported to form an orderly regulation network for plant-biotic stress interactions, improving plant tolerance to adverse environments. In particular, SA chiefly mediated acquired plant resistance, whereas JA and ET mediated induced systemic resistance (ISR) in response to biotic stresses [31]. ABA has been reported to affect plant responses to biotic stress mainly via interaction with other stress response pathways [32]. In the present study, many up-regulated genes were observed to be involved in plant hormone metabolism and signal transduction pathways, mainly ABA and JA pathways.

ABA is considered a negative regulatory factor in plant disease resistance, and its expression is associated with increased disease sensitivity [32], [33]. In the ABA signal transduction pathway, there are three core factors, ABA receptor PYR/PYL/RCAR protein (the most upstream regulator in the ABA signaling pathway), protein phosphatase (PP2C, negative regulatory factor) and SNF1-related protein kinase 2 (SnRK2, positive regulatory factor). These three factors form a double negative regulatory system to regulate ABA signal transduction and its downstream reactions. After the plant produces ABA induced by growth and development signals or environmental stimuli, ABA binds to PYR/PYL/RCAR protein, and interacts with PP2C to inhibit protein phosphatase activity as well as removes PP2C inhibition on SnRK2, thereby activating the ABA signal response [34]. Studies suggest that (Table 8), after *S. scitamineum* infection, TDFs of PYR/PYL, PP2C and SnRK2 are differentially expressed, indicating that the ABA signaling pathway is involved in the response of sugarcane to *S. scitamineum*. After 48 and 120 h of sugarcane and *S. scitamineum* interaction, in “ROC”22, sustained up-regulation of SnRK2 TDFs (log_2_FC = 1.43 and 1.53, respectively) was detected. In comparison, after 48 h of sugarcane and *S. scitamineum* interaction, in Yacheng05-179 one down-regulated SnRK2 TDF (log_2_FC = −1.10) was found. Thus, after the susceptible cultivar is infected by the pathogen, high expression of SnRK2 may activate the ABA signal, making it susceptible to infection or facilitating the reproduction and spread of the pathogen after infection.

When plants respond to environmental stress, signaling molecule JA acts the most rapidly, playing an important role in resistance reactions [23]. Studies indicate that after biotic and abiotic stresses, JA-related gene expression is up-regulated, causing JA accumulation [23]. MYC2 belongs to the myelocytomatosis protein family (MYCs), and is an MYC transcription factor with a role in regulating the JA response pathway and directly regulating downstream response genes. Jasmonate ZIM-Domain (JAZ) protein is a major inhibitory factor in the JA signaling pathway, able to bind to SCFcOI protein, leading to degradation mediated by 26S proteasome [23]. In *Arabidopsis thaliana* the COIl-JAZs-MYC2 complex function has been elucidated-when *A. thaliana* is not stimulated by JA type hormones, JAZ protein binds to MYC2, preventing MYC2 binding to downstream genes and JA response gene activation. When JA type hormones stimulate the plant, JA type signaling molecules activate the SCFCOIl protein complex, which competes with binding of JAZ to MYC2, releasing MYC2 proteins that bind to JA response genes, ultimately regulating plant physiology [35]. As Table 8 shows, after *S. scitamineum* infection of sugarcane, JAZ and MYC2 genes were up-regulated suggesting that *S. scitamineum* can stimulate the JA biosynthesis and that the JA signaling pathway is involved in the response of sugarcane to *S. scitamineum*. In addition, as infection time prolonged, differentially expressed TDFs of JAZ and MYC2 increased as did their expression in Yacheng05-179 (more so than in “ROC”22), suggesting that in Yacheng05-179 JA signaling pathway activation in response to *S. scitamineum* is stronger.

#### Flavonoid Biosynthesis Pathway

Flavonoids are secondary metabolites widely present in plants with important roles in many biological processes (including response to biotic and abiotic stresses) [36], [37]. The phenylpropanoid metabolic pathway is the key metabolic pathway leading to the flavonoid pathway. Under the catalysis of phenylalanine ammonia lyase (PAL), phenylpropanoid produces cinnamic acid; then after catalysis of cinnamate-4 hydroxylase (C4H), cinnamic acid produces 4-coumaric acid which then yields 4-coumarate CoA under the catalysis of 4-coumarate CoA ligase (4CL). Next, under the catalysis of chalcone synthase (CHS) and chalcone isomerase (CHI), 4-coumarate CoA and its derivatives enter the downstream flavonoid biosynthetic pathway [38]. PAL, C4H and 4CL are key enzymes in the phenylpropanoid metabolic pathway, with roles in plant responses to various biotic abiotic stresses including pathogen infection, mechanical damage and exogenous hormone stimuli [23].

Here, we observed that (Table 8) *S. scitamineum* induced up-regulation of PAL, C4H, 4CL genes in both Yacheng05-179 and “ROC”22, yet there were more differentially expressed TDFs in Yacheng05-179 (PAL: sugar cane_unigene_BMK.40935, sugar cane_unigene_BMK.51492 and gi35076956; C4H: sugar cane_unigene_BMK.74288, sugar cane_unigene_BMK.65142 and gi35122896; 4CL: sugar cane_unigene_BMK.57158 and gi35030858) than in “ROC”22 (PAL: gi34918942; C4H: sugar cane_unigene_BMK.45497). These data show that resistance-associated genes involved in the flavonoid biosynthesis pathway in the resistant cultivar exceed those in the susceptible cultivar, and that sugarcane can synthesize polyphenolic compounds with antibacterial effects to defend itself from *S. scitamineum*.

#### Plant-pathogen Interaction Pathway

In long-term interactions with pathogens, plants form a series of defense mechanisms [39] and these include phytoalexin (PA) formation, hypersensitive response (HR) production, enzyme changes (such as peroxidase and polyphenol oxidase) and accumulation of pathogenesis-related proteins [40]. At 24, 48 and 120 h after *S. scitamineum* infection, in “ROC”22 proportions of differentially expressed genes involved in plant-pathogen interactions were affected (See Table 6). In Yacheng05-179, these proportions were also noted (Table 7), suggesting that the defense response occurs earlier than 48 h.

In addition, we observed that under *S. scitamineum* stress, pathogenesis-associated genes in sugarcane were expressed differentially (Table 8), including 10 glucanase, 26 chitinase genes and 1 catalase gene. Catalase is an important antioxidant enzyme in plants, functioning to clear and protect against metabolically produced H_2_O_2_. Studies suggest that catalase is needed for plant defense, stress response, and regulation of the cellular redox balance [41], [42]. After 48 and 120 h of sugarcane and *S. scitamineum* interaction, transcription and expression of the catalase gene was induced. Chitinase and β-1,3-glucanase are two typical plant pathogenesis-associated proteins documented to act synergistically in defense against pathogenic fungi. Under normal condition, expression of chitinase and β-1,3-glucanase in plants is relatively low. After pathogenic fungal stress, β-1,3-glucanase and chitinase defense proteins accumulate in the cells. Currently, β-1,3-glucanase and chitinase have been detected in nearly 100 plant species, and β-1,3-glucanase and chitinase genes in many plants have been cloned [43]. Reports indicate that successful enhancement of plant resistance to diseases can be accomplished by introduction of exogenous β-1,3-glucanase and chitinase genes. In *O. sativa, Triticum aestivum, Nicotiana tabacum* and other species, trans-chitinase and/or trans-β-1,3-glucanase gene plants have been obtained [44], [45]. Chitinases were encoded by a multi-gene family which have been reported to group into seven classes (Class I–VII) [46], [47]. Thokoane and Rutherford investigate differentially expressed genes after sugarcane exposure to *S. scitamineum* and sequence homology analysis revealed that chitinase protein family members were induced after *S. scitamineum* infection for 7 d [7]. RT-qPCR showed a high gene expression level of a sugarcane class IV chitinase gene *ScChiB1* (EU914815.1) in the resistant cultivar than in the susceptible one during interaction with *Colletotrichum falcatum* [48]. Chitinases have been shown to inhibit the growth of chitin-containing fungi, both *in* *vitro* [49], [50] and *in* *vivo* [51], [52]. Our present study revealed that chitinases were triggered during *S. scitamineum* infection and data from inoculation experiments and the validation of *in* *vitro* antibacterial activity suggest a close relationship between the expression of *ScChi* and plant immunity. These data indicate that the sugarcane chitinase gene identified through RNA-Seq analysis is relevant to plant-pathogen interaction.

#### Cell Wall Fortification Pathway

The cell wall maintains plant cell morphology and participates in physiological activities such as extracellular signal identification [53]. Fungi obtain nutrition via saprophytes, parasitosis or symbiosis and *Ustilago maydis* in *Z. mays* and *Blumeria graminis f. sp. Hordei* in *Triticum* aestivum are documented to be biotrophic pathogens. In their genomes, genes encoding cell wall degradation enzymes are fewer than that in saprophytic fungi. Typically, these two fungi do not directly degrade plant cell walls; instead, they form haustoria in host epidermal cells to absorb nutrition [54], [55]. Studies suggest that the cell wall is the first line of plant defense against pathogens [56], [57]. After pathogen stress, with cell wall damage, disease signaling pathways are activated to initiate cell wall defense reactions. For example, cell walls surrounding invading pathogens produce and accumulate callose, phenolics and lignin, increasing the strength of the cell wall [58]. Genes encoding attachment protein receptors such as syntaxin in *Hordeum vulgare, N. tabacum* and *A. thaliana* participate in cell wall fortification, improving plant resistance [59]. We observed that (Table 8), after *S. scitamineum* inoculation, expression of genes involved in cell wall fortification were up-regulated in sugarcane, including 8 syntaxin genes and 9 hydroxyproline-rich glycoprotein (HRGP) genes. Also, 120 h after *S. scitamineum* inoculation, expression of waxy gene CER1 (gi36041011) was down-regulated (log_2_FC = −1.63) in “ROC”22, but remained unchanged in Yacheng05-179. Likely, increased expression of genes encoding proteins positively associated with the cell wall may enhance the resistance of sugarcane to *S. scitamineum*, and genes encoding proteins negatively associated with the cell wall may have the opposite effect. Thus, differential expression of these genes between resistant and susceptible sugarcane cultivars reflects cultivar resistance and susceptibility.

#### Resistance-associated Transcription Factors

Plant disease resistance involves coordinated expression of defense response genes. Transcriptional regulation commands expression of plant defense response genes, altering plant susceptibility or resistance and this is mediated by transcription factors [60]. When a plant is infected, signal transduction transcription factors in the plant are activated, and these then interact with the corresponding cis-acting elements via DNA-protein interaction, triggering expression of relevant defense response genes [61]. Studies suggest that transcription factors associated with plant disease resistance mainly include MYB transcription factor, the WRKY family in zinc finger proteins and ERF-type transcription factor [61].

MYB transcription factors can participate in plant systemic acquired resistance (SAR) and HR. Previously, over-expression of *A. thaliana* AtMYB30 in *A. thaliana* and *N. tabacum* were reported to result in HR to different pathogenic bacteria, and resistance to many bacterial diseases was enhanced [62]. *A. thaliana* MYB protein BOS1 can regulate plant resistance to *Botrytis cinerea*, *Alternaria alternata* and other necrotizing pathogens [63]. The WRKY family is a class of zinc finger proteins present in higher plants and all family members contain 1 to 2 WRKY (WRKYGQK) conserved domains. WRKY family proteins can bind the highly conserved element W-box in promoters of many plant defense response genes, offering important roles in plant defense responses [61]. Over-expression of the *O. sativa* WRKY45 gene can increase the resistance of *O. sativa* to *Pyricularia oryzae Cav.* [64]. *A. thaliana* WRKY1l and WRKY17 have negative regulatory roles in its basic resistance [61], and WRKY18, WRKY40 and WRKY60 can form complexes regulating plant disease resistance [65]. ERF proteins are members of the plant AP2/EREBP transcription factor superfamily and previously many ERF transcription factors were isolated from *A. thaliana, O. sativa, N. tabacum, T. aestivum* and other plants, and their roles in plant growth and development, as well as in response to biotic and abiotic stresses, have been documented [66]. Here, we found (Table 8) that in *S. officinarum* there were 25 MYB, 18 WRKY and 18 ERF differentially expressed genes. Compared to the susceptible cultivar, the number of activated transcription factors in the resistant cultivar was higher, and there were more up/down regulation of transcripts. These data suggest that the above-mentioned transcription factors actively regulate sugarcane resistance to *S. scitamineum*.

## Conclusion

In summary, with RNA-Seq techniques, we performed transcriptome analysis on sugarcane genotypes at different resistance levels at different time points after *S. scitamineum* infection. Data indicate that as infection time was prolonged, activated differentially expressed genes in sugarcane increased. Differentially expressed genes induced by *S. scitamineum* inoculation in the resistant Yacheng05-179 cultivar and the susceptible “ROC”22 cultivar were similar. However, overall the expression time of resistance-associated genes in Yacheng05-179 (24–48 h) was earlier than that in “ROC”22 (48–120 h), and more transcript expressions were observed in the former, suggesting resistance specificity and early timing of these genes in non-affinity interactions between sugarcane and *S. scitamineum*. Data regarding potential functions of sugarcane TDFs in response to *S. scitamineum* will lay the foundation for future investigations into the role of these candidate genes in sugarcane-*S. scitamineum* interactions, and inform genomic studies on sugarcane smut resistance.

## Supporting Information

File S1Contains the following files: **Table S1.** Primers used for RT-qPCR analysis of differentially expressed genes. **Table S2.** Gene ontology classification of up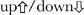 regulated genes in “ROC”22 after *S. scitamineum* inoculation. **Table S3.** Gene ontology classification of 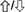 regulated genes in Yacheng05-179 after *S. scitamineum* inoculation.(DOC)
