# Correction: A Global View of Transcriptome Dynamics during *Sporisorium scitamineum* Challenge in Sugarcane by RNA-seq

**DOI:** 10.1371/journal.pone.0118445

**Published:** 2015-02-06

**Authors:** The PLOS ONE Staff

In the Funding section, the grant number for the Fujian Province University grant is incorrect. The correct grant number is: JA14095.
