# Supplementary material for: A Global View of Transcriptome Dynamics during Sporisorium scitamineum Challenge in Sugarcane by RNA-seq
Source: PLoS One. 2014 Aug 29;9(8):e106476. doi: 10.1371/journal.pone.0106476 (PMC4149577; doi:10.1371/journal.pone.0106476)
Supplement: File S1 — Contains the following files: Table S1. Primers used for RT-qPCR analysis of differentially expressed genes. Table S2. Gene ontology classification of up regulated genes in “ROC”22 after S. scitamineum inoculation. Table S3. Gene ontology classification of regulated genes in Yacheng05-179 after S. scitamineum inoculation. (DOC) [file pone.0106476.s001.doc]

**Supporting Information**

Table S1. Primers used for RT-qPCR analysis of differentially expressed genes

| **Gene name** | **Gene ID** | **Forward primer (5’-3’)** | **Reverse primer (5’-3’)** |
| --- | --- | --- | --- |
| Q1 | Sugarcane_Unigene_BMK.40387 | ACGGGTTCTGGCAGTGGGA | GGCATCATGGCGAAGTTGTTT |
| Q2 | Sugarcane_Unigene_BMK.49302 | ATGCGGTGCCCTTTGATT | TTGGTCCAGTAGTGGTTGATGTT |
| Q3 | Sugarcane_Unigene_BMK.51436 | GTTGGCAGCATCAGGCAGTT | TGTCCAGGTCCAGGAAATCG |
| Q4 | Sugarcane_Unigene_BMK.57924 | CGTGCTATGATGGCTGATTTG | TCGTTGGGAGGTTCTCGTTT |
| Q5 | Sugarcane_Unigene_BMK.63074 | ACAGAACATTTGCGAAGCAGTG | GCGTTGGTGAGGTTGAGGC |
| Q6 | Sugarcane_Unigene_BMK.63784 | CCTCAACCTCCACCTTCACC | CTGGGCCTGCGTTCCTT |
| *GAPDH* | — | CACGGCCACTGGAAGCA | TCCTCAGGGTTCCTGATGCC |

**Table S2. Gene ontology classification of up⇧/down⇩ regulated genes in "ROC"22 after *S. scitamineum* inoculat**ion

| **GO classification** | **T2 *vs.* T1** | | **T3 *vs.* T1** | | **T4 *vs.* T1** | |
| --- | --- | --- | --- | --- | --- | --- |
|  | **⇧regulated** | **⇩regulated** | **⇧regulated** | **⇩regulated** | **⇧regulated** | **⇩regulated** |
| cell part | 256 | 105 | 411 | 123 | 1,000 | 394 |
| cell | 253 | 104 | 402 | 121 | 984 | 391 |
| organelle | 232 | 103 | 367 | 118 | 919 | 383 |
| cellular process | 226 | 94 | 373 | 109 | 884 | 357 |
| metabolic process | 219 | 80 | 363 | 97 | 861 | 315 |
| binding | 213 | 80 | 325 | 92 | 773 | 329 |
| response to stimulus | 172 | 64 | 274 | 79 | 710 | 248 |
| biological regulation | 159 | 59 | 225 | 72 | 571 | 267 |
| catalytic activity | 132 | 43 | 247 | 61 | 603 | 186 |
| organelle part | 116 | 56 | 176 | 60 | 358 | 202 |
| developmental process | 108 | 33 | 161 | 50 | 393 | 202 |
| membrane | 103 | 65 | 198 | 75 | 521 | 163 |
| cellular component organization or biogenesis | 99 | 39 | 171 | 53 | 358 | 227 |
| multi-organism process | 89 | 18 | 133 | 29 | 343 | 70 |
| localization | 82 | 53 | 154 | 56 | 384 | 142 |
| establishment of localization | 73 | 50 | 145 | 51 | 363 | 127 |
| reproduction | 72 | 38 | 102 | 39 | 275 | 144 |
| reproductive process | 71 | 36 | 101 | 38 | 273 | 136 |
| multicellular organismal process | 65 | 29 | 111 | 42 | 289 | 169 |
| extracellular region | 52 | 23 | 80 | 28 | 191 | 71 |
| signaling | 49 | 26 | 60 | 32 | 208 | 83 |
| membrane-enclosed lumen | 43 | 10 | 48 | 14 | 114 | 73 |
| macromolecular complex | 43 | 31 | 56 | 30 | 85 | 22 |
| immune system process | 36 | 22 | 50 | 30 | 163 | 51 |
| membrane part | 33 | 19 | 65 | 28 | 171 | 55 |
| growth | 33 | 9 | 57 | 13 | 136 | 56 |
| nucleic acid binding transcription factor activity | 30 | 4 | 23 | 7 | 72 | 29 |
| cell junction | 27 | 10 | 48 | 12 | 128 | 45 |
| viral reproduction | 13 | 4 | 19 | 4 | 29 | 9 |
| transporter activity | 10 | 13 | 38 | 15 | 107 | 104 |
| structural molecule activity | 8 | 11 | 16 | 9 | 17 | 15 |
| electron carrier activity | 8 | 2 | 16 | 1 | 48 | 10 |
| death | 7 | 6 | 11 | 8 | 47 | 18 |
| extracellular region | 6 | 13 | 15 | 13 | 24 | 20 |
| antioxidant activity | 6 | 3 | 10 | 5 | 18 | 3 |
| receptor activity | 5 | 1 | 4 | 3 | 31 | 6 |
| enzyme regulator activity | 5 | 3 | 10 | 5 | 19 | 9 |
| synapse | 3 | 0 | 3 | 1 | 2 | 1 |
| protein binding transcription factor activity | 3 | 0 | 0 | 0 | 1 | 5 |
| molecular transducer activity | 3 | 10 | 5 | 11 | 38 | 19 |
| pigmentation | 3 | 1 | 9 | 3 | 13 | 16 |
| rhythmic process | 3 | 2 | 8 | 1 | 12 | 2 |
| synapse | 2 | 0 | 2 | 0 | 1 | 0 |
| cell proliferation | 2 | 5 | 2 | 7 | 7 | 33 |
| extracellular matrix | 1 | 12 | 2 | 13 | 3 | 13 |
| extracellular matrix part | 1 | 1 | 1 | 2 | 0 | 0 |
| locomotion | 1 | 4 | 3 | 7 | 13 | 8 |
| translation regulator activity | 0 | 0 | 2 | 0 | 0 | 0 |
| biological adhesion | 0 | 0 | 0 | 2 | 7 | 3 |
| channel regulator activity | 0 | 0 | 0 | 0 | 0 | 1 |
| chemoattractant activity | 0 | 0 | 0 | 0 | 0 | 1 |
| nutrient reservoir activity | 0 | 0 | 0 | 0 | 0 | 1 |
| cell killing | 0 | 0 | 0 | 0 | 9 | 0 |

**Table S3. Gene ontology classification of up⇧/down⇩ regulated genes in Yacheng05-179 after *S. scitamineum*** inoculation

| **GO classification** | **T6 *vs.* T5** | | **T7 *vs*. T5** | | **T8 *vs.* T5** | |
| --- | --- | --- | --- | --- | --- | --- |
|  | **⇧regulated** | **⇩regulated** | **⇧regulated** | **⇩regulated** | **⇧regulated** | **⇩regulated** |
| cell | 412 | 151 | 666 | 167 | 554 | 164 |
| cell part | 412 | 151 | 668 | 167 | 561 | 165 |
| metabolic process | 380 | 125 | 609 | 149 | 487 | 147 |
| organelle | 373 | 146 | 619 | 158 | 507 | 159 |
| cellular process | 367 | 138 | 611 | 155 | 489 | 153 |
| binding | 306 | 115 | 544 | 136 | 434 | 129 |
| response to stimulus | 300 | 100 | 458 | 112 | 377 | 96 |
| biological regulation | 251 | 100 | 406 | 105 | 329 | 95 |
| catalytic activity | 247 | 73 | 410 | 97 | 291 | 98 |
| membrane | 204 | 55 | 354 | 66 | 295 | 80 |
| organelle part | 172 | 60 | 302 | 75 | 242 | 85 |
| cellular component organization or biogenesis | 146 | 57 | 276 | 52 | 180 | 47 |
| developmental process | 143 | 67 | 279 | 66 | 214 | 58 |
| localization | 141 | 44 | 252 | 57 | 194 | 60 |
| multicellular organismal process | 125 | 51 | 230 | 55 | 172 | 48 |
| establishment of localization | 124 | 39 | 217 | 50 | 172 | 55 |
| multi-organism process | 122 | 35 | 208 | 39 | 165 | 31 |
| extracellular region | 103 | 22 | 122 | 26 | 107 | 22 |
| reproduction | 97 | 46 | 173 | 47 | 147 | 36 |
| reproductive process | 94 | 47 | 168 | 47 | 142 | 37 |
| membrane part | 83 | 18 | 148 | 22 | 115 | 35 |
| signaling | 76 | 33 | 133 | 37 | 100 | 28 |
| immune system process | 49 | 12 | 87 | 15 | 61 | 11 |
| growth | 47 | 11 | 87 | 10 | 64 | 7 |
| membrane-enclosed lumen | 46 | 10 | 83 | 18 | 68 | 14 |
| macromolecular complex | 46 | 17 | 99 | 24 | 70 | 38 |
| cell junction | 41 | 15 | 72 | 17 | 47 | 14 |
| transporter activity | 40 | 7 | 68 | 12 | 58 | 21 |
| nucleic acid binding transcription factor activity | 29 | 19 | 52 | 23 | 57 | 17 |
| enzyme regulator activity | 23 | 5 | 14 | 7 | 11 | 6 |
| electron carrier activity | 19 | 5 | 34 | 10 | 31 | 12 |
| extracellular region | 17 | 3 | 18 | 4 | 16 | 2 |
| viral reproduction | 13 | 3 | 18 | 3 | 16 | 2 |
| antioxidant activity | 9 | 0 | 18 | 0 | 18 | 1 |
| death | 9 | 7 | 22 | 10 | 20 | 7 |
| molecular transducer activity | 7 | 10 | 22 | 9 | 14 | 7 |
| structural molecule activity | 6 | 1 | 10 | 3 | 8 | 6 |
| cell killing | 6 | 1 | 1 | 1 | 1 | 1 |
| pigmentation | 6 | 0 | 13 | 1 | 8 | 0 |
| receptor activity | 5 | 2 | 12 | 3 | 13 | 2 |
| extracellular matrix | 4 | 1 | 3 | 1 | 3 | 1 |
| cell proliferation | 4 | 2 | 8 | 1 | 2 | 0 |
| biological adhesion | 4 | 1 | 4 | 0 | 4 | 0 |
| protein binding transcription factor activity | 3 | 1 | 7 | 3 | 6 | 3 |
| synapse | 2 | 0 | 5 | 0 | 2 | 0 |
| locomotion | 2 | 3 | 9 | 3 | 2 | 1 |
| extracellular matrix part | 1 | 0 | 4 | 0 | 2 | 0 |
| synapse | 1 | 0 | 4 | 0 | 2 | 0 |
| rhythmic process | 1 | 12 | 9 | 12 | 8 | 13 |
| translation regulator activity | 0 | 1 | 0 | 1 | 0 | 0 |
| nutrient reservoir activity | 0 | 1 | 0 | 1 | 0 | 1 |
| chemoattractant activity | 0 | 0 | 0 | 1 | 0 | 0 |
| receptor regulator activity | 0 | 0 | 0 | 0 | 1 | 0 |
| nucleoid | 0 | 0 | 0 | 0 | 0 | 1 |
| channel regulator activity | 0 | 0 | 0 | 0 | 0 | 1 |
